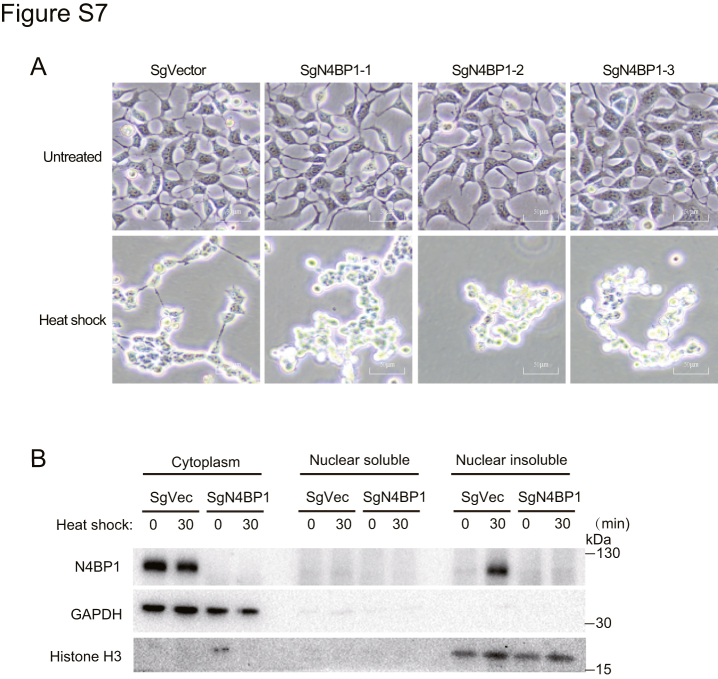# N4BP1 is a nucleocytoplasmic shuttling protein and recognizes aggregates of the ubiquitin-like protein NEDD8 to protect cells under heat shock

**DOI:** 10.1016/j.jbc.2025.110511

**Published:** 2025-07-21

**Authors:** Xiaohong Guo, Yuhan Ke, Yuanmeng Chen, Yanli Li, Jie Zhang, Wen Zheng, Peida Feng, Yihan Ji, Zitao Wang, Yang Lu, Renfang Mao, Yihui Fan

**Affiliations:** 1 Department of Pathogenic Biology, School of Medicine, Nantong University, Nantong China; 2 Laboratory of Medical Science, School of Medicine, Nantong University, Nantong, China; 3 Department of Pediatric Surgery, Affiliated Hospital of Nantong University, Nantong, China; 4 The Intensive Care Unit, Affiliated Hospital of Nantong University, Nantong, China; 5 Department of Pathophysiology, School of Medicine, Nantong University, Nantong, China

**Keywords:** N4BP1, nucleocytoplasmic shuttling, aggregates, NEDD8, heat shock

## Abstract

N4BP1 (NEDD4-binding partner 1) is a key checkpoint for proper inflammatory responses; however, its cellular localization, biologic nature, and functions in other cellular processes remain largely unknown. In this study, we demonstrate that N4BP1 is a nucleocytoplasmic shuttling protein. Treatment with leptomycin B induces nuclear accumulation of N4BP1, and the region responsible for its nuclear distribution maps to amino acids 151 to 338. Further analysis identified a nuclear localization signal (NLS) spanning residues 279 to 299. Deletion or mutation of this NLS abolishes N4BP1 nuclear import, while fusing the NLS to GFP is sufficient to drive GFP into the nucleus. Notably, we found that N4BP1 forms protein aggregates in both the cytoplasm and nucleus. These aggregates lack ubiquitin-modified proteins but instead colocalize with NEDD8-modified proteins. Consistently, N4BP1 aggregates contain cullin-1 and cullin-2. The CoCUN domain is essential for recognizing neddylated proteins and mediating N4BP1 aggregate formation. N4BP1 aggregates exhibit liquid–liquid phase separation, as evidenced by their sensitivity to 1,6-hexanediol (an liquid–liquid phase separation inhibitor). Smaller N4BP1 aggregates can fuse into larger one and reassemble after 1,6-hexanediol-induced disruption. Furthermore, heat shock promotes N4BP1 aggregation, which confers cellular protection under stress conditions. Taken together, our findings reveal that N4BP1 is a nucleocytoplasmic shuttling protein. N4BP1 forms protein aggregates that contain neddylated proteins such as cullin-1 and cullin-2. This study uncovers the previously unrecognized role of N4BP1 in organizing neddylated protein aggregates and highlights its functional significance in stress adaption.

N4BP1 (NEDD4-binding partner 1) was initially identified in a screen for NEDD4-interacting proteins and has been shown to inhibit the function of ITCH (1, 2). Recent studies have revealed important roles for N4BP1 in both physiological and pathological conditions (3, 4, 5). In inflammatory responses, N4BP1 negatively regulates cytokine expression and is cleaved by caspase-8 (3, 4). During virus infection, it directly degrades viral RNA and undergoes cleavage by MALT1 (6). Additionally, N4BP1 suppresses NF-kB signaling by inhibiting NEMO oligomerization through its UBA and CoCUN domains (7). Beyond these domains, N4BP1 contains KH and NYN domains, which enable it to degrade mRNA substrates *via* coding sequences (8). However, the precise function of these individual domains and their regulation remains poorly defined. Initially, N4BP1 was reported to localize to the nucleus, particularly the nucleolus (9). Nevertheless, more recent studies describe it as a cytoplasmic protein, leaving its true cellular distribution unresolved (3). Thus, the cellular distribution of N4BP1 and how various domains cooperate to play fundamental role is unclear. Furthermore, given its UBA and CoCUN domains (which are associated with ubiquitin system), whether N4BP1 plays a role in proteostasis has yet to be determined.

Proteostasis is achieved through a complex network of pathways that maintain proper protein synthesis, folding, trafficking, disaggregation, and degradation of approximately 20,000 proteins within and around a cell (10, 11). It is essential for cell health, growth, survival and organismal development, and stress adaptation. A decline or failure in proteostasis can lead to aging, diseases, and stress-associated injuries (10, 11). The ubiquitin system serves as the primary defense mechanism for proteostasis and has been extensively studied (12). In contrast, the role of NEDD8 (an ubiquitin-like protein) in proteostasis remains unclear, despite its emerging importance in aging and age-related proteinopathies such as Alzheimer’s disease (13, 14). Temperature fluctuations are among the most common stresses, and heat shock occurs when the temperature rises above the cell’s optimal range (15). The well studied cellular response to heat shock called heat shock response, which is a transcriptional program that induces the expression of chaperones and cytoprotective factors to enhance the refolding of damaged proteins (16). Beyond transcriptional regulation, protein aggregates have also been shown to contribute to heat shock survival (17, 18). Although small heat shock protein was identified in protein aggregates dynamically, the components and the regulatory mechanisms are still largely undetermined (19).

Neddylation is a posttranslational modification involving the covalent attachment of the ubiquitin-like protein NEDD8 to substrates *via* lysine residues (20). Abnormal neddylation is associated with various human diseases, including neurodegenerative disorders, cancers, atherosclerosis, chronic liver diseases, heart disease, autoimmune diseases, and obesity (21, 22, 23). The most common protein substrates for neddylation are cullins, but non-cullin proteins also reported to be neddylated recently (24). Neddylation of non-cullin proteins could affect their stability, activity, and subcellular localization. For instance, MDM2-mediated neddylation of P53 inhibits its transcriptional activity (25). Polyneddylated proteins could also form aggregates under stress conditions (26), but the function of NEDD8-containing aggregates and their recognition mechanisms remain largely unknown.

Major biological processes, such as DNA replication and protein synthesis, occur in distinct cellular compartments: the nucleus and the cytoplasm, respectively (27). Since the first nucleocytoplasmic shuttling protein was identified, numerous other proteins including transcription factors and RNA-binding proteins have been found to shuttle between the nucleus and cytoplasm (28, 29). Alterations in the subcellular distribution of nucleocytoplasmic shuttling protein are associated with cancer and neurodegenerative diseases (30, 31). Therefore, identifying novel nucleocytoplasmic shuttling proteins is important for understanding their regulatory mechanisms and their roles in disease.

## Results

### N4BP1 is a shuttling protein between cytoplasma and nuclear insoluble fraction

**Figure 1:**
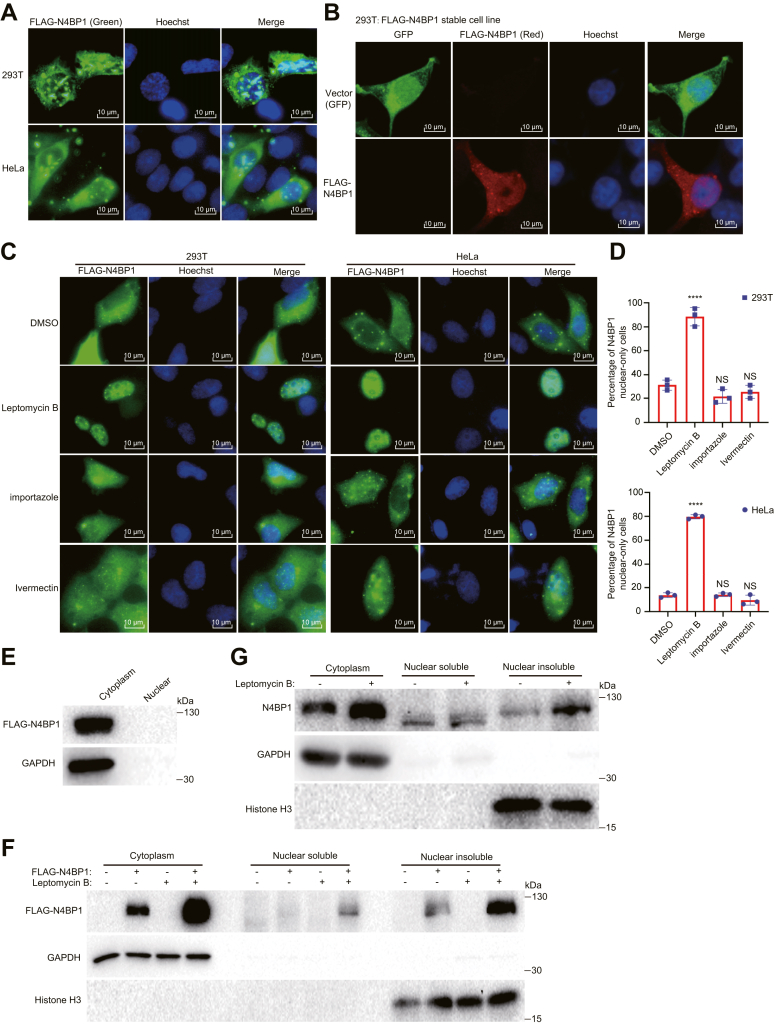
**N4BP1 shuttles between cytoplasma and nuclear insoluble fraction.***A*, 293T and HeLa cells were transfected with plasmids encoding FLAG-N4BP1, and the distribution of N4BP1 was determined by immunofluorescence using an anti-FLAG antibody. The images were acquired using a fluorescence microscope. Scale bar is 10 μm. *B*, the distribution of FLAG-N4BP1 in control and stably expressed 293T cells were determined by immunofluorescence using an anti-FLAG antibody. The images were acquired using a confocal microscope. Scale bar is 10 μm. *C*, 293T and HeLa cells were transfected with plasmids encoding FLAG-N4BP1, followed by treatment with 10 μM leptomycin B, importazole, or ivermectin for 1 h. The distribution of N4BP1 was determined by immunofluorescence using an anti-FLAG antibody. The images were acquired using a fluorescence microscope. Scale bar is 10 μm. *D*, quantification of N4BP1 nuclear-only cells in (*C*). N4BP1 nuclear-only cells were counted from 30 cells. *Top*: The radio of N4BP1 nuclear-only cells in FLAG-N4BP1-expressing 293T cells before and after drug treatment. *Bottom*: The radio of N4BP1 nuclear-only cells in FLAG-N4BP1-expressing HeLa cells before and after drug treatment. ∗∗∗∗*p* < 0.0001. *E*, 293T cells were transfected with plasmids encoding FLAG-N4BP1. The distribution of N4BP1 was determined by WB after biochemical fractionation of cytoplasm and nuclear extracts. *F*, HeLa cells were transfected with plasmids encoding FLAG-N4BP1 and then treated with 10 μM leptomycin B for 24 h. The distribution of N4BP1 was determined by WB after nuclear and cytoplasmic fractionation and insoluble component extraction. *G*, HeLa cells were treated with 10 μM leptomycin B for 24 h. The distribution of N4BP1 was determined by WB after nuclear and cytoplasm fractionation and insoluble component extraction. Data in (*A* and *E*) are representative of four independent experiments. Data in (*B* and *C*, *F* and *G*) are representative of three independent experiments. N4BP1, NEDD4-binding partner 1.

**Supplemental Figure S1:**
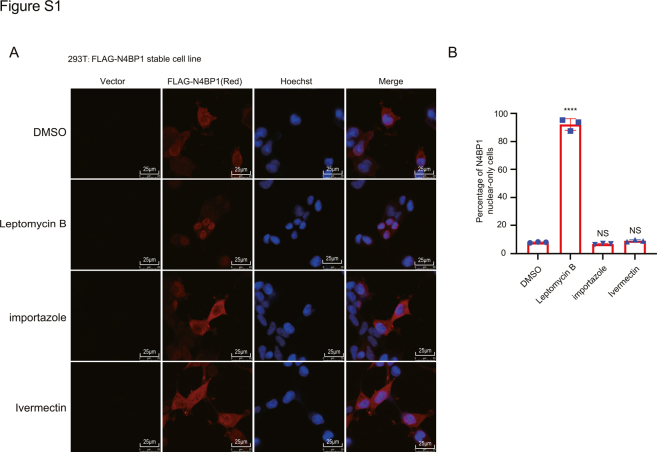


To explore the cellular distribution of N4BP1, we expressed FLAG-N4BP1 in 293T cells. As shown, N4BP1 localized to both the cytoplasm and the nucleus (Fig. 1*A*). A similar distribution was observed in HeLa cells (Fig. 1*A*). To further confirm this result, we established FLAG-N4BP1 stable-expressing cell lines, in which FLAG-N4BP1 also exhibited dual localization (Fig. 1*B*). To examine whether N4BP1 is a shuttling protein between the cytoplasm and nucleus, we used leptomycin B to inhibit the nuclear export. Notably, leptomycin B treatment significantly block the nuclear export of N4BP1, resulting in nuclear accumulation of N4BP1 both in 293T and HeLa cells (Fig. 1, *C* and *D*). The same effect was observed in FLAG-N4BP1 stable-expressing cell lines (Fig. S1, *A* and *B*). Thus, our results suggest that N4BP1 is a shuttling protein between the cytoplasm and nucleus. To further validate this conclusion, we conducted biochemical fractionation of the cytoplasm and nucleus. Surprisingly, we did not detect FLAG-N4BP1 in the nuclear soluble fraction, despite a clear nuclear distribution of FLAG-N4BP1 under fluorescence microscopy (Fig. 1*E*). It suggests that FLAG-N4BP1 is in the nuclear insoluble fraction. Therefore, we collected the insoluble fraction of the nucleus. Indeed, we observed FLAG-N4BP1 in insoluble fraction of the nucleus, and its amount was increased by leptomycin B treatment (Fig. 1*F*). To assess whether our findings are relevant to physiological conditions, we detected endogenous N4BP1 in cellular fractions. Consistently, endogenous N4BP1 also localized to the cytoplasm and nuclear insoluble fraction, the amount of which was increased by leptomycin B treatment (Fig. 1*G*). Together, our results clearly demonstrate that N4BP1 is a shuttling protein between the cytoplasm and nucleus. Within the nucleus, N4BP1 is insoluble.

### A domain spanning residues 151–338aa is required for the nuclear import of N4BP1

**Figure 2:**
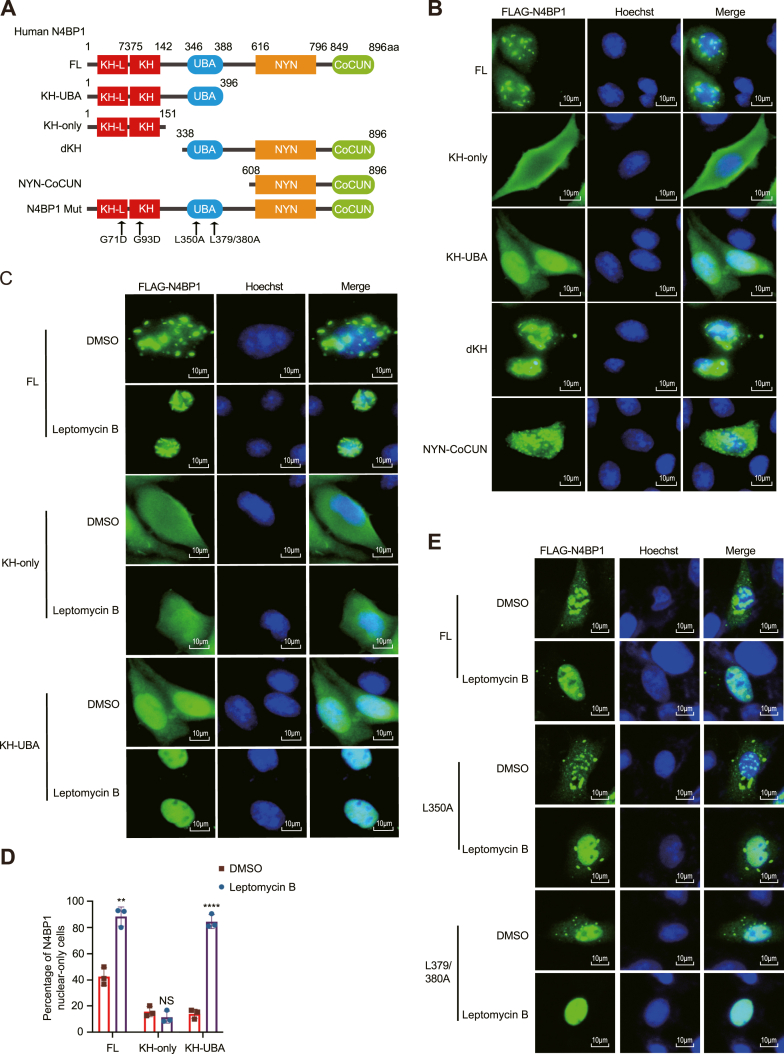
**N4BP1 nuclear import relies on a domain between amino acids 151 to338.***A*, schematic representations of full-length N4BP1, N4BP1 deletion mutants, and N4BP1 point mutants. *B*, HeLa cells were transfected with plasmids encoding FLAG-tagged full-length N4BP1 or its deletion mutants. N4BP1 localization was determined by immunofluorescence using an anti-FLAG antibody. The images were acquired using a fluorescence microscope. Scale bar is 10 μm. *C*, HeLa cells were transfected with plasmids encoding FLAG-N4BP1, KH-only, or KH-UBA were treated with 10 μM leptomycin B for 4 h. N4BP1 localization was acquired using immunofluorescence (anti-FLAG). Images were captured with a fluorescence microscope. Scale bar is 10 μm. *D*, quantification of N4BP1 nuclear-exclusive localization in (*C*). Thirty FLAG-N4BP1-positive HeLa cells were counted. The ratio of cells displaying nuclear-only N4BP1 before and after leptomycin B treatment is shown. ∗∗*p* < 0.01; ∗∗∗∗*p* < 0.0001. *E*, HeLa cells were transfected with FL-N4BP1, N4BP1-L350A, or N4BP1-L379/380A and then treated with 10 μM leptomycin B for 4 h. N4BP1 distribution was assessed by immunofluorescence (anti-FLAG). Images were obtained using a confocal microscope. Scale bar is 10 μm. Data in (*B*, *C*, and *E*) are representative of three independent experiments. N4BP1, NEDD4-binding partner 1.

**Supplemental Figure S2:**
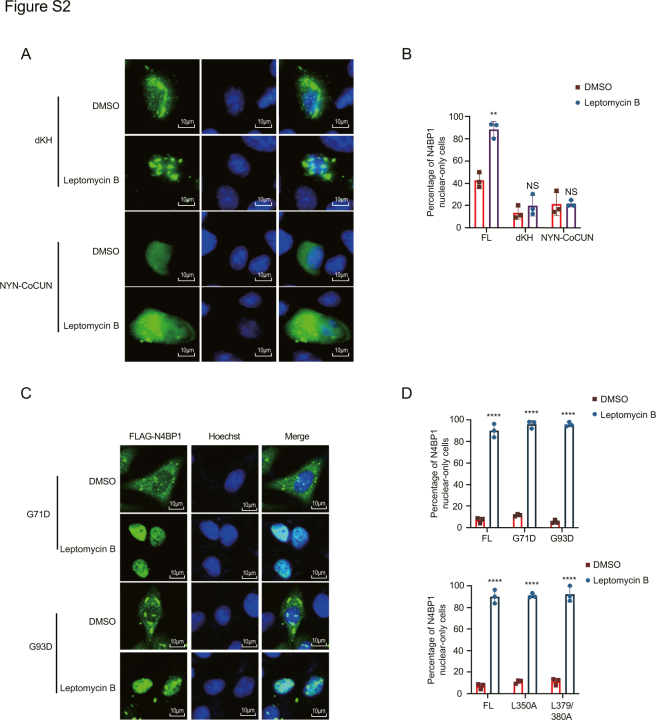


Having established that N4BP1 resides in the nucleus, we investigated which domain is essential for its nuclear localization. N4BP1 consists of four domains: KH, UBA, NYN, and CoCUN (Fig. 2*A*). The KH-only domain was exclusively cytoplasmic. However, inclusion of the 151–396aa region (containing the UBA domain) resulted in dual localization to both the cytoplasm and nucleus (Fig. 2*B*). It suggests that the region between residues 151–396aa mediates the nuclear distribution of N4BP1. Consistent with this, mutants lacking the 151–338aa (dKH and NYN-CoCUN) predominantly reside in the cytoplasm (Fig. 2*B*). Treatment with Leptomycin B leads to nuclear accumulation of FL and KH-UBA mutants but has minimal impact on KH-only, dKH, and NYN-CoCUN mutants (Fig. 2, *C* and *D*, Fig. S2, *A* and *B*). Point mutants (L350A, L379/380A) that disrupt the UBA domain function do not affect N4BP1 distribution (Fig. 2, *A* and *E* and Fig. S2*D*) (32). Similarly, mutants G71D and G93D, which impair the KH domain function, also had no effect on N4BP1 distribution (Fig. 2*A*, Fig. S2, *C* and *D*) (33). Our findings indicate that the region responsible for mediating N4BP1 nuclear distribution is located between residues 151–338aa.

### Identifying a nuclear localization signal from amino acids 279 to 299

**Figure 3:**
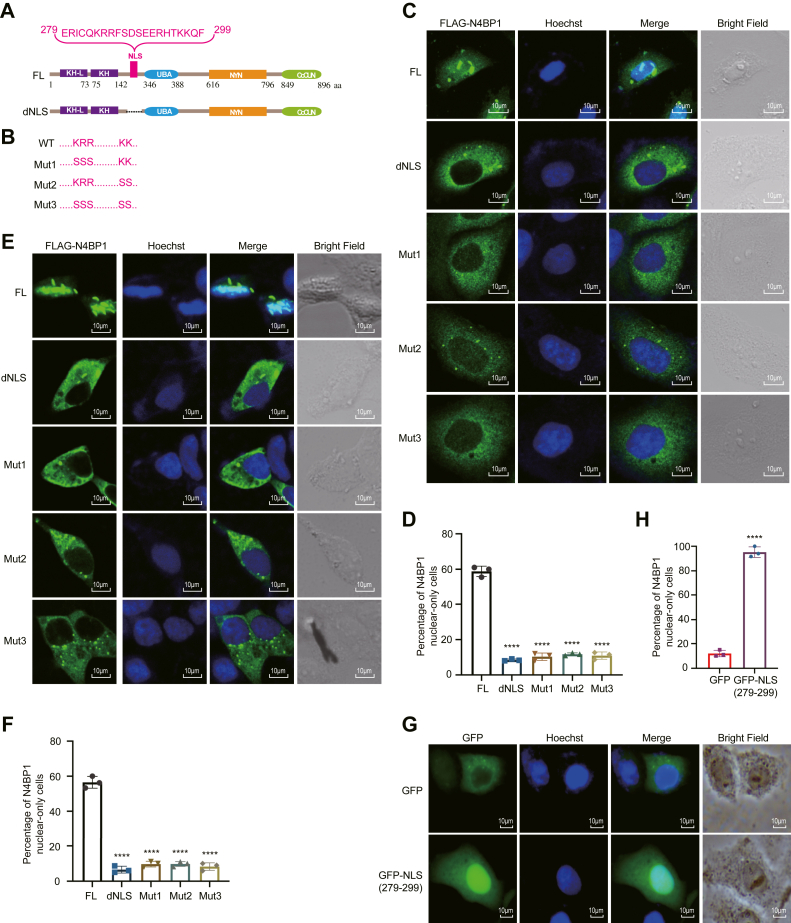
**The NLS (279-299aa) of N4BP1 is important for its nuclear distribution.***A*, schematic diagrams of full-length N4BP1 and its NLS-deleted mutant (dNLS; residues 279-299: ERICQKRRFSDSEERHTKKQF). *B*, schematics of wildtype N4BP1 and NLS region mutants (Mut1-3). *C*, HeLa cells transfected with FL-N4BP1, dNLS, Mut1, Mut2, or Mut3 were analyzed by immunofluorescence (anti-FLAG). Images were observed by a confocal microscope. Scale bar is 10 μm; *D*, quantification of nuclear-exclusive N4BP1 in (*C*). Thirty FLAG-N4BP1-positive HeLa cells were counted. The ratio of nuclear-only cells posttransfection is shown. ∗∗∗∗*p* < 0.0001. *E*, 293T cells transfected with FL-N4BP1, dNLS, Mut1, Mut2, or Mut3 were analyzed by immunofluorescence (anti-FLAG). Images were observed by a confocal microscope. Scale bar is 10 μm; *F*, quantification of nuclear-exclusive N4BP1 in E. Thirty FLAG-N4BP1-positive 293T cells were counted. ∗∗∗∗*p* < 0.0001. *G*, HeLa cells transfected with GFP or GFP-NLS (279-299) were imaged by a fluorescence microscope. Scale bar is 10 μm. *H*, quantification of nuclear-exclusive GFP-NLS localization in (*G*). Thirty GFP-positive cells were counted. ∗∗∗∗*p* < 0.0001. Data in (*C*, *E*, and *G*) are representative of three independent experiments. N4BP1, NEDD4-binding partner 1; NLS, nuclear localization signal.

**Supplemental Figure S3:**
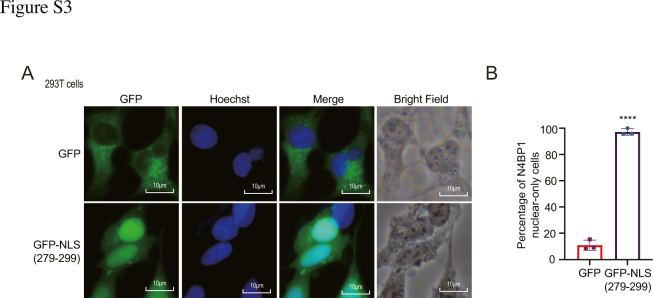


The transport of proteins from the cytoplasm into the nucleus is mediated by nuclear localization signals (NLSs). Therefore, we utilized the NLS prediction program (https://nls-mapper.iab.keio.ac.jp/cgi-bin/NLS_Mapper_form.cgi) to identify potential NLS in human N4BP1. A putative NLS ranging from amino acids 279 to 299 (ERICQKRRFSDSEERHTKKOF) was predicted (Fig. 3*A*). To validate the function of the predicted NLS, we constructed plasmids encoding a mutant N4BP1 with an NLS deletion. Additionally, we introduced point mutations to analyze the key amino acid of the NLS (Fig. 3*B*). As depicted in Figure 3*C*, full-length (FL) N4BP1 is located in both the cytoplasm and nucleus in HeLa cells (Fig. 3*C*). However, the NLS deletion mutant is solely located at cytoplasm. The point mutations (KRR to SSS or KK to SS) also result in localization to the cytoplasm (Fig. 3, *C* and *D*). In 293T cells, we obtained similar results, with deletion of the NLS or its point mutants leading to exclusive cytoplasmic localization (Fig. 3, *E* and *F*). These findings clearly demonstrate that the NLS is essential for the nuclear distribution of N4BP1. To further emphasize the importance of the identified NLS, we fused it to the C terminus of GFP (Fig. 3*G* and Fig. S3). As illustrated, wildtype GFP primarily resides in the cytoplasm, whereas GFP fused with the NLS is predominantly located in the nucleus both in HeLa and 293T cells (Fig. 3, *G* and *H*, Fig. S3, *A* and *B*). In summary, our results indicate that the 279–299aa NLS is both necessary and sufficient for the nuclear distribution of N4BP1.

### N4BP1 forms phase-separated aggregates and its nuclear distribution is excluded from DNA

**Figure 4:**
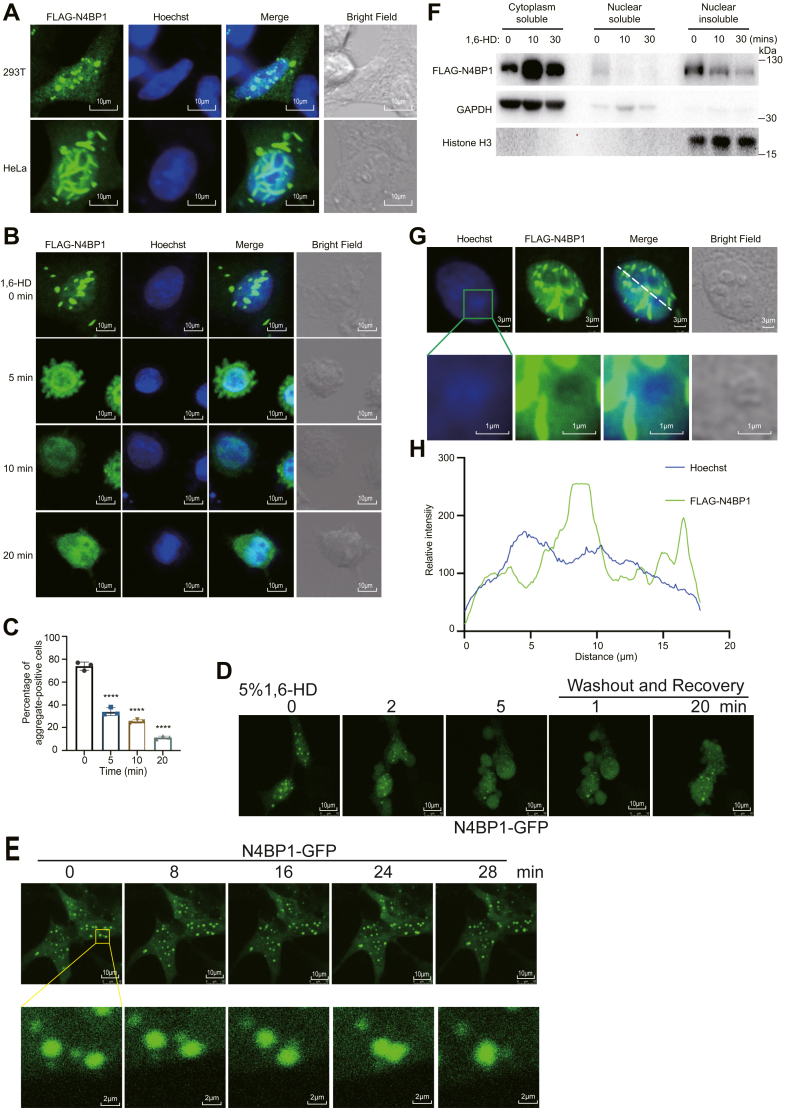
**N4BP1 can form aggregates and N4BP1 distribution is excluded with DNA at nucleus.***A*, 293T and HeLa cells were transfected with plasmids encoding FLAG-N4BP1 and the aggregates of N4BP1 were determined by immunofluorescence (anti-FLAG). The images were observed by a confocal microscope. Scale bar is 10 μm. *B*, HeLa cells were transfected with plasmids encoding FLAG-N4BP1 and then treated with 3% of 1,6-hexanediol for 0, 5, 10, or 20 min. The aggregates of N4BP1 were determined by immunofluorescence (anti-FLAG). The images were observed by a confocal microscope. Scale bar is 10 μm; *C*, quantification of aggregate-positive cells in (*B*). Fifty FLAG-N4BP1-positive cells were counted. The ratio after 1,6-HD treatment is shown. ∗∗∗∗*p* < 0.0001. *D*, time-lapse imaging of N4BP1-GFP aggregate dissolution and recovery in 293T cells treated with 5% 1,6-HD. Scale bar is 10 μm. *E*, time-lapse imaging of N4BP1-GFP aggregates in 293T cells. Scale bar is 10 μm (*top*) or 2 μm (*bottom*). *F*, HeLa cells were transfected with plasmids encoding FLAG-N4BP1 and then treated with 3% of 1,6-hexanediol for 0, 10, or 30 min. The distribution of N4BP1 was determined by WB after nuclear and cytoplasmic fractionation assays and insoluble component extraction. *G*, HeLa cells were transfected with plasmids encoding FLAG-N4BP1, and the aggregates of N4BP1 was determined at nucleus by immunofluorescence using antibody against FLAG. The images were observed by a confocal microscope. Scale bar is 3 μm (*top*) or 1 μm (*bottom*). *H*, relative fluorescence intensity at the *white dotted line* (*G*) was quantified by using Image J. Data in (*A*) are representative of five independent experiments. Data in (*B*, *D*–*G*) are representative of three independent experiments. N4BP1, NEDD4-binding partner 1; 1,6-HD, 1,6-hexanediol.

**Supplemental Figure S4:**
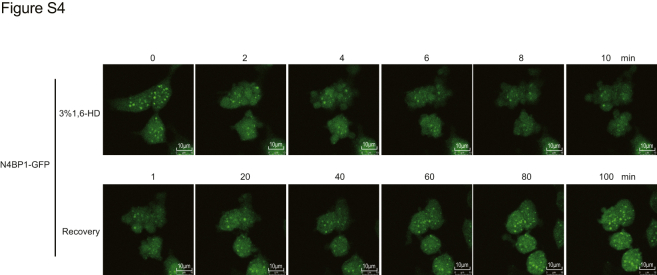


Alongside the observation of the nuclear distribution of N4BP1, we consistently observed that N4BP1 forms aggregates in both the cytoplasm and the nucleus (Fig. 4*A*). These N4BP1 aggregates can be observed in both 293T and HeLa cells. It is known that protein aggregates can form through either solid or liquid–liquid phase separation. To distinguish the nature of N4BP1 aggregates, we treated cells with 1,6-hexanediol (1,6-HD) that is an inhibitor to disrupt liquid–liquid phase separation (Fig. 4*B*). As demonstrated, 1,6-HD treatment significantly disrupted the N4BP1 aggregates in both the cytoplasm and nucleus (Fig. 4, *B* and *C*). To monitor the N4BP1 aggregates in living cells without staining, we fused N4BP1 with GFP. N4BP1-GFP also form aggregates in living cells (Fig. 4*D* and Fig. S4). The N4BP1-GFP aggregates were dissolved within 5 min by treatment with 3% or 5% 1,6-HD and recovered in 20 min after 1,6-HD removal (Fig. 4*D* and Fig. S4). These results indicate that N4BP1 aggregates are liquid–liquid phase separation. This conclusion was further supported by that small N4BP1-GFP aggregates could fusion into a large one (Fig. 4*E* and Movie M1). N4BP1 aggregates are insoluble because that 1,6-HD treatment decreased the amount of N4BP1 in the insoluble fraction (Fig. 4*F*). These results suggest that the N4BP1 aggregates form through liquid–liquid phase separation, and they are less soluble. Within the nucleus, we overlapped the fluorescence from N4BP1 and DNA (Hoechst). Interestingly, N4BP1 distribution is excluded from DNA (Fig. 4, *G* and *H*). It implies that N4BP1 may colocalize with RNA in the nucleus. Together, these results indicate that N4BP1 can form an insoluble fraction through aggregation *via* liquid–liquid phase separation.

### N4BP1 aggregates contain NEDD8-modified but not ubiquitin-modified proteins

**Figure 5:**
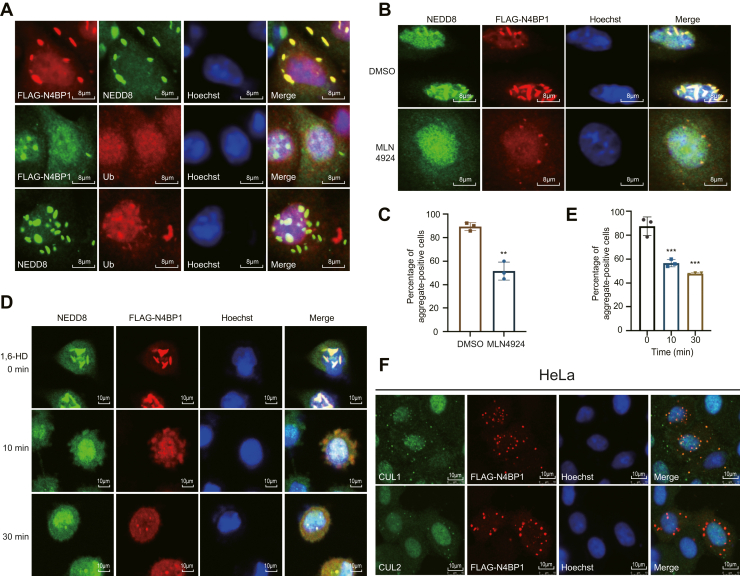
**N4BP1 recognizes NEDD8-modifiedproteins to form aggregates.***A*, HeLa cells were transfected with plasmids encoding FLAG-N4BP1. *Top*: The immunofluorescence of NEDD8 (*green*) costained with FLAG-N4BP1 (*red*); *Middle*: The immunofluorescence of FLAG-N4BP1 (*green*) costained with Ub (*red*); *Bottom*: The immunofluorescence of NEDD8 (*green*) costained with Ub (*red*). The images were observed by a confocal microscope. Scale bar is 8 μm. *B*, HeLa cells expressing FLAG-N4BP1 were treated with 2 μM MLN4924 for 24 h. The immunofluorescence of NEDD8 (*green*) costained with FLAG-N4BP1 (*red*), and the images were observed by a confocal microscope. Scale bar is 10 μm. *C*, quantification of aggregate-positive cells in (*B*). Twenty FLAG-N4BP1-positive cells were counted. ∗∗*p* < 0.01. *D*, HeLa cells expressing FLAG-N4BP1 were treated with 3% of 1,6-hexanediol for 0, 10, or 30 min. The immunofluorescence of NEDD8 (*green*) costained with FLAG-N4BP1 (*red*), and the images were observed by a confocal microscope. Scale bar is 10 μm. *E*, quantification of aggregate-positive cells in (*D*). Twenty cells were counted. ∗∗∗*p* < 0.001 *F*, HeLa cells were transfected with plasmids encoding FLAG-N4BP1. The immunofluorescence of CUL1 and CUL2 (*green*) costained with FLAG-N4BP1 (*red*), and the images were observed by a confocal microscope. Scale bar is 10 μm. Data in (*A*) are representative of five independent experiments. Data in (*B*, *D*, and *F*) are representative of three independent experiments. N4BP1, NEDD4-binding partner 1.

**Supplemental Figure S5:**
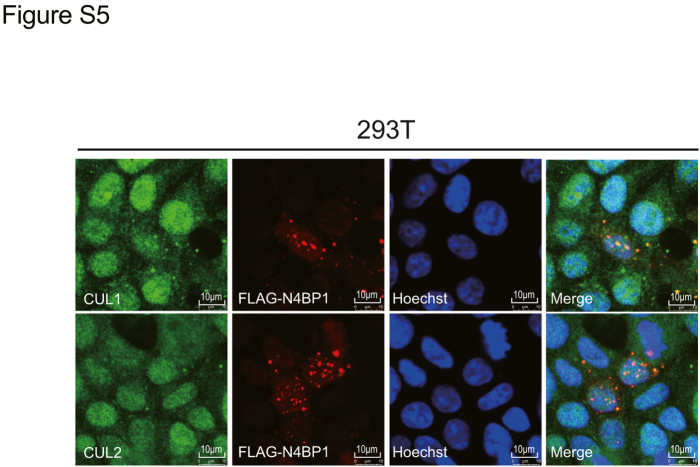


It has been suggested that N4BP1 might recognize ubiquitin. Therefore, we tested whether N4BP1 aggregates contain ubiquitinated proteins. However, immunofluorescence analysis revealed no colocalization between ubiquitin staining and N4BP1 aggregates (Fig. 5*A*). It suggests that N4BP1 aggregates do not contain ubiquitinated proteins. Interestingly, we found that N4BP1 aggregates overlap well with NEDD8 staining (Fig. 5*A*). These results suggested that N4BP1 aggregates contain neddylated but not ubiquitinated proteins. To further investigate whether N4BP1 aggregates are associated with NEDD8 modification, we used the small-molecule inhibitor MLN4924 to block NEDD8 modification. As shown, MLN4924 treatment greatly disrupts N4BP1 aggregates (Fig. 5, *B* and *C*). Additionally, the LLPS inhibitor 1,6-HD also markedly reduced N4BP1 aggregates containing neddylated proteins (Fig. 5, *D* and *E*). Because that cullin proteins are major substrates of neddylation in cells, we asked whether N4BP1 aggregates contain cullins. As shown, N4BP1 aggregates overlap well with cullin-1 aggregates in HeLa cells (Fig. 5*F*). Cullin-2 staining shows similar results with Cullin-1 (Fig. 5*F*). Parallel experiments in 293T cells confirmed these findings, showing N4BP1 aggregates containing both cullin-1 and cullin-2 (Fig. S5). Together, our results suggest that N4BP1 recognizes NEDD8-modified proteins including its major substrates, cullin-1 and cullin-2, to form aggregates.

### The CoCUN domain mediates NEDD8 recognition and N4BP1 aggregate formation

**Figure 6:**
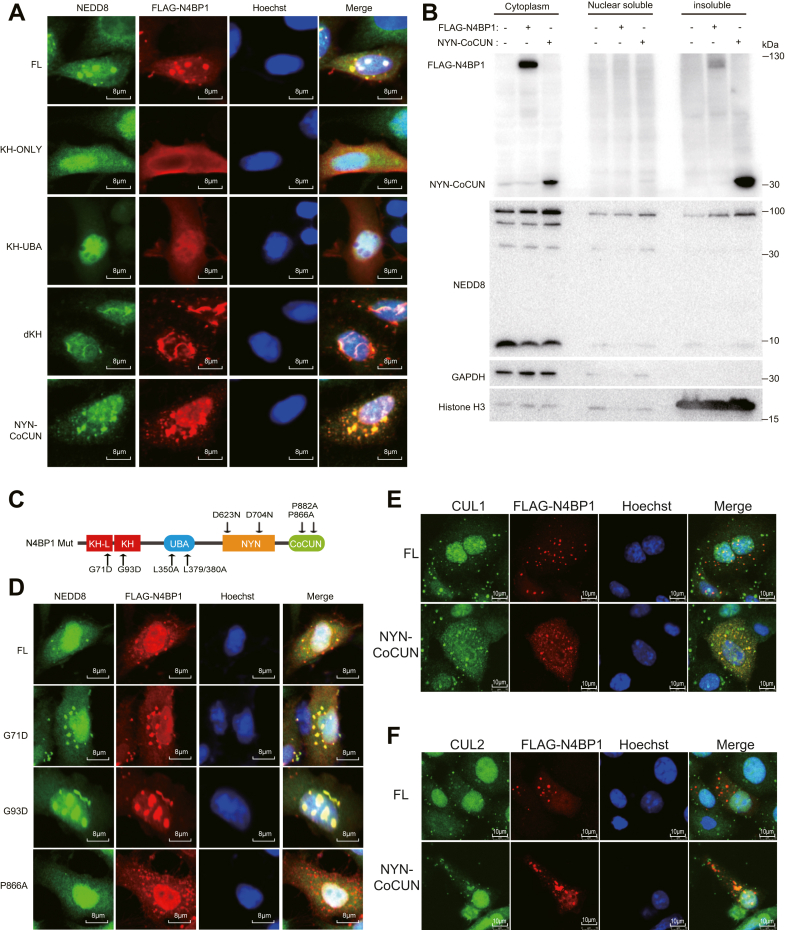
**The P866 of CoCUN domain is required for N4BP1 to recognized NEDD8.***A*, HeLa cells were transfected with plasmids encoding FLAG-N4BP1 or different N4BP1 deletion mutants. The immunofluorescence of NEDD8 (*green*) costained with FLAG-N4BP1 (*red*), and the images were observed by a confocal microscope. Scale bar is 8 μm. *B*, 293T cells were transfected with plasmids encoding FLAG-N4BP1 or NYN-CoCUN. The distribution of N4BP1 and NEDD8 were determined by WB after nuclear and cytoplasmic fractionation assays and insoluble component extraction. *C*, schematic representations of N4BP1 point mutants in KH, UBA, NYN, and CoCUN domains. *D*, HeLa cells were transfected with plasmids encoding FLAG-N4BP1-FL, G71D, G93D, or P866A. The immunofluorescence of NEDD8 (*green*) costained with FLAG-N4BP1 (*red*), and the images were observed by a confocal microscope. Scale bar is 8 μm. *E*, HeLa cells were transfected with plasmids encoding FLAG-N4BP1 or NYN-CoCUN. The immunofluorescence of CUL1 (*green*) costained with FLAG-N4BP1 (*red*), and the images were observed by a confocal microscope. Scale bar is 10 μm. *F*, HeLa cells were transfected with plasmids encoding FLAG-N4BP1 or NYN-CoCUN. The immunofluorescence of CUL2 (*green*) costained with FLAG-N4BP1 (*red*), and the images were observed by a confocal microscope. Scale bar is 10 μm. Data in (*A*, *D*–*F*) are representative of three independent experiments. Data in (*B*) are representative of two independent experiments. N4BP1, NEDD4-binding partner 1.

To understand the molecular mechanism underlying N4BP1 aggregates, we examined the domain necessary for this function. As shown, KH-only and KH-UBA domains failed to form N4BP1 aggregates (Fig. 6*A*). However, CoCUN domain-containing mutants, including dKH and NYN-CoCUN, exhibit comparable or enhanced aggregation than the FL protein (Fig. 6*A*). Consistent with its cytoplasmic localization (due to lacking the 279-299 NLS), NYN-CoCUN primarily formed cytoplasmic aggregates (Fig. 6*A*). Consistent with N4BP1 aggregates are insoluble, NYN-CoCUN dramatically increased in the insoluble fraction (Fig. 6*B*). For wildtype N4BP1, majority of protein are in the soluble fraction. However, majority of NYN-CoCUN protein are in insoluble fraction (Fig. 6*B*). These results suggest that CoCUN domain is crucial for forming N4BP1 aggregates. To further confirm these findings, we performed point mutations to disrupt each domain (Fig. 6*C*). Consistently, the P866A mutant, which disrupts the function of CoCUN domain, also impair the formation of N4BP1 aggregates (Fig. 6*D*). Although small N4BP1 aggregates were observed under P866A mutant, these N4BP1 aggregates do not colocalized with NEDD8. In contrast, other mutants such as G71D, G93D, L350A, L379/380A, D623N, D704N, and P882A showed normal aggregation (Fig. 6*D*) (6, 7, 32, 33, 34). These results indicate that P866 is essential for N4BP1 to recognize NEDD8 and subsequently form large aggregates. Like wildtype N4BP1, NYN-CoCUN-induced N4BP1 aggregates also contain cullin-1 and cullin-2 (Fig. 6, *E* and *F*). Together, the formation of N4BP1 aggregates is dependent on its CoCUN domain, which recruits neddylated proteins such as cullin-1 and cullin-2.

### N4BP1 aggregates play a protective role in cellular response to heat shock

**Figure 7:**
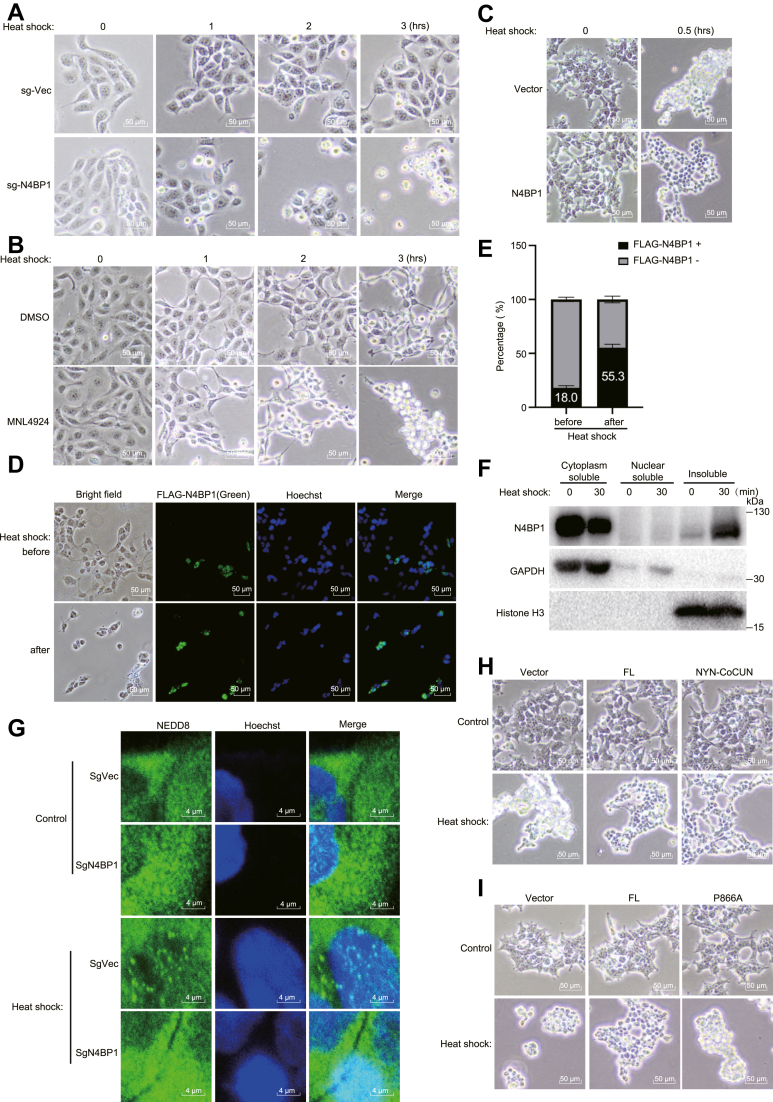
**N4BP1/NEDD8 aggregates play important role in heat shock.***A*, control and N4BP1 stably knocking down HaCaT cells were heated at 42 °C, and cell death was monitored hourly for 3 h by bright-field microscopy. Scale bar is 50 μm. *B*, control and N4BP1 stably knocking down HaCaT cells were treated with 2 μM MLN4924 for 6 h and then were treated in 42 °C. Cell death was monitored hourly for 3 h by bright-field microscopy. Scale bar is 50 μm. *C*, control and N4BP1-overexpression 293T cells were heated at 42 °C for 0.5 h, and cell death was observed by an optical microscope. Scale bar is 50 μm. *D*, 293T cells were transfected with plasmids encoding FLAG-N4BP1 and then heated at 42 °C for 20 min. Cell death was determined by immunofluorescence (anti-FLAG). The images were observed by a fluorescence microscope. Scale bar is 50 μm. *E*, quantification analysis of FLAG-N4BP1-positive cells in (*D*). One hundreds of cells from three different fields were counted. The ratio of FLAG-N4BP1-positive cells before and after heat shock were presented. *F*, 293T cells were treated or untreated in 42 °C for 30 min. The distribution of N4BP1 was determined by WB after nuclear and cytoplasmic fractionation assays and insoluble component extraction. *G*, control and N4BP1 stably knocking down HaCaT cells were heated at 42 °C for 2 h, and the aggregates of NEDD8 were determined by immunofluorescence (anti-NEDD8). The images were observed by a confocal microscope. Scale bar is 4 μm. *H*, 293T cells were transfected with plasmids encoding FLAG-N4BP1 or NYN-CoCUN and then heated at 42 °C for 0.5 h. Cell death was observed by an optical microscope. Scale bar is 50 μm. *I*, 293T cells were transfected with plasmids encoding FLAG-N4BP1 or P866A mutant and then heated at 42 °C for 20 min. Cell death was observed by an optical microscope. Scale bar is 50 μm. Data in (*A*–*D*, *F*–*I*) are representative of three independent experiments. N4BP1, NEDD4-binding partner 1.

Protein aggregates are believed to play a significant role during stress responses, such as heat shock. To investigate whether N4BP1 aggregates are crucial in this context, we subjected control and N4BP1-KO cells to a time-course heat shock experiments. We observed that N4BP1-deficient cells are significantly more sensitive to heat shock compared to control cells (Fig. 7*A*). Consistent with the observation that N4BP1 aggregates contain NEDD8 proteins, we noted that cells treated with MLN4924 also exhibited increased sensitivity to heat shock (Fig. 7*B*). Similar results were observed in 293T cells (Fig. S7*A*). Furthermore, overexpression of N4BP1 was found to protect cells from heat shock (Fig. 7*C*). To further validate the critical role of N4BP1 in heat shock, we transfected FLAG-N4BP1 in 293T cells. Prior to heat shock, approximately 18% of the cells were positive for FLAG-N4BP1. However, after heat shock, over 50% of the surviving cells exhibited FLAG-N4BP1 positivity (Fig. 7, *D* and *E*). In summary, our results suggest that N4BP1 plays a protective role in cells under heat shock conditions.

### Manipulation of N4BP1 aggregates alters cellular response to heat shock

Given the crucial role of N4BP1 aggregates in heat shock, we investigated whether manipulating these aggregates would change the cellular response to heat shock. Heat shock induces significant translocation of N4BP1 into the insoluble fraction (Fig. 7*F* and Fig. S7*B*). During heat shock, the number of N4BP1/NEDD8 aggregates increases (Fig. 7*G*). Notably, heat shock-induced NEDD8 aggregates are greatly reduced in N4BP1-deficient cells (Fig. 7*G*). The NYN-CoCUN mutant, which exhibits enhanced aggregation capacity, demonstrates a stronger protective effect on cells compared to the wildtype N4BP1 (Fig. 7*H*). Although, ectopic expression of N4BP1 protects cells from heat shock, the aggregation-deficient P866A mutant completely lost this protective capacity (Fig. 7*I*). In summary, our results demonstrated that N4BP1 aggregates play important role in heat shock.

## Discussion

The trafficking of many proteins to the nucleus is mediated by several well-studied karyopherins, for instance, importin-β (IMPβ) through its cargo adaptor IMPα (35). IMPα recognizes two subclasses of classical NLS, including monopartite cNLS (consensus: K-K/R-X-K/R) and bipartite cNLS (consensus: K/R-K/R-X10–12-K/R3/5). Through domain deletion, we mapped the NLS region from amino acids 151 to 396 as responsible for the nuclear distribution of N4BP1. Within this region, we identified a potential NLS (amino acids 279-299: ERICQKRRFSDSEERHTKKQF), which is a bipartite cNLS. In this region, the KRR/KK sequence is critical for N4BP1’s entry into the nucleus, as mutations to these residues completely disrupt the nuclear localization of N4BP1. This region is located between the KH and UBA domains. Adjacent to this region, there are two caspase 8 cleavage sites. Although the cNLS recognized by IMPα, the nuclear distribution is unaffected by importazole and ivermectin, two inhibitors targeting IMPβ or IMPα/IMPβ complex, respectively. It suggests that the nuclear translocation of N4BP1 is an IMPα dependent but IMPβ independent pathway. In previous reports, CaMKIV is transported to the nucleus using IMPα but without utilizing IMPβ (36, 37). Thus, it is intriguing to explore the relationship between the cNLS-mediated N4BP1 nuclear translocation and the function of different member of karyopherins.

N4BP1 is a member of the NYN domain family, which includes seven members: ZC3H12A (Regnase-1), ZC3H12B, ZC3H12C, ZC3H12D, KHNYN, NYNRIN, and N4BP1 (38, 39). In previous report, KHNYN was found to contain a newly identified domain called the Cullin-binding domain associated with NEDD8 (CUBAN) (40). CUBAN is located at the carboxyl end of KHNYN and has a strong preference for NEDD8. At the carboxyl end of N4BP1, there is also a CoCUN (849-896aa) domain, a newly identified cousin domain of CUBAN (32). However, in their *in vitro* testing system, the CoCUN domain lacks the NEDD8-binding properties as observed in CUBAN. Despite this, we observed a clear overlap between N4BP1 aggregates and NEDD8. Furthermore, the P866A mutant, which disrupts the function of CoCUN, also reduces the colocalization between NEDD8 and N4BP1. In the N4BP1 aggregates, we did not observe ubiquitin, suggesting that N4BP1 aggregates contain neddylated but not ubiquitinated proteins. This is further supported by the fact that the NEDD8 inhibitor MLN4924 greatly reduces the formation of N4BP1 aggregates. Furthermore, N4BP1 aggregates contain cullin-1 and cullin-2, two major substrates of neddylation in cells. Therefore, unlike *in vitro* conditions, the CoCUN domain might favor NEDD8 during the formation of N4BP1 aggregates *in vivo*. But, how the specificity of the CoCUN domain toward NEDD8 is achieved *in vivo* remains unknown.

Upon experiencing heat shock, proteins form aggregate to initiate heat shock response or to aid cellular adaptation to thermal stress (41, 42). These aggregates can be distinguished by their nature; some may be more liquid-like, while others are more solid-like, each with distinct functions (43). A portion of these aggregates consists of denatured and misfolded proteins due to sudden increase in temperature, while others are regulatory biomolecular condensates comprised of ribosomal proteins and stress granule components (44, 45). In certain cells, N4BP1 forms large aggregates under ectopic conditions. These N4BP1 aggregates are liquid-like because they are easily disrupted by the inhibitor 6-HD. Thus, they are more likely to be regulatory condensates that adapt the cell to heat shock. Interestingly, these N4BP1 condensates are colocalized with NEDD8 but not with ubiquitin. Heat shock promotes neddylation and thus protects the ubiquitin proteasome system (46). However, reported neddylation under heat shock involves mixed chains of NEDD8 and ubiquitin, mediated by ubiquitin E1 enzyme Ube1 rather than the NEDD8-activating enzyme (20, 47). However, our reported N4BP1 aggregates contain only NEDD8 and play a protective role under heat shock. It is still largely unclear how N4BP1 specifically recruits NEDD8-modified proteins but not ubiquitinated or mixed chains.

In summary, we provide evidence to show N4BP1 is a nucleocytoplasmic shuttling protein. The amino acids from 279 to 299 serve as NLSs that facilitating its nuclear distribution. N4BP1 also forms aggregates that play a protective role under heat shock. Our results demonstrate several novel nature of N4BP1, including its nuclear distribution and liquid-like aggregates. These novel aspects of N4BP1 provide new insights into understanding its biological functions.

## Experimental procedures

### Cell culture, transfection, and drug treatment

293T, HeLa, and HaCaT cells were cultured in high-sugar Dulbecco's modified Eagle’s medium (DMEM) (Cytiva, SH30243.01) supplemented with 10% fetal bovine serum (EallBIo, 03.U16001DC) and 1% penicillin–streptomycin in a 37 °C incubator with 5% CO2. Plasmid transfection was performed using Lipofectamine 2000 (Invitrogen) according to the manufacturer’s instructions. Drugs were added after cells adhesion and that included importazole (Selleck, S8446), ivermectin (Selleck, S1351), leptomycin B (Selleck, S7580), MLN4924 (APExBIO, B1036) and 1,6-HD (Sigma-Aldrich, 629-11-8).

### Plasmids

The coding sequences of human N4BP1 were cloned into FLAG-pRK or pEnCMV-3×FALG expression vectors. N4BP1 deletion mutants were constructed in FLAG-pRK, including 1 to 151 (KH-only), 1 to 396 (KH-UBA), 338 to 896 (dKH), and 608 to 896 (NYN-CoCUN). Site-directed mutants were generated in pEnCMV-N4BP-3×FLAG: G71D, G93D, L350A, L379 380A, D623N, D704N, P866A, and P882A. The amino acid sequence of the N4BP1 NLS (279-299) is ERICQKRRFSDSEERHTKKQF. Mut1 mutation of the NLS changes KRR to SSS and Mut2 mutation changes KK to SS. Mut3 combines both mutations (RKK→SSS and KK→SS). All sequences were verified by DNA sequencing.

### Immunofluorescence

Cells were seeded in 24-well plates with coverslips (WHB Scientific, WHB-24-CS) and then cultured in 37 °C incubator. After transfection, when cell density reached 70% confluency, cells were fixed with 4% paraformaldehyde (Biosharp Biotechnology, BL539A) for 1 h at room temperature, followed by three PBS washes. Cells were permeabilized with 0.1% TritonX-100 (Sangon Biotech, A600198-0500) in PBS for 10 min and blocked in 1% BSA (Biosharp Biotechnology, BS114) for 30 min at room temperature. Primary antibodies were incubated overnight at 4 °C. The next day, cells were washed five times with PBS and incubated with fluorescent secondary antibodies for 1h at room temperature. Nuclei were stained with Hoechst 33342 (Beyotime Biotechnology, C1022) for 1 h at room temperature. After five PBS washes, coverslips were mounted on glass slides using anti-fade mounting medium (Beyotime Biotechnology, P0126-5ml), sealed with nail polish, and cured in the dark for 1 h. All images were acquired using a fluorescence microscope (Leica, DM5000B) and a confocal microscope (Leica, TCSSP8).

### Nuclear and cytoplasmic fractionation and insoluble component extraction

Nuclear and cytoplasmic fractions were separated using a nuclear-cytoplasmic protein preparation kit (Applygen Technologies, P1200) according to the manufacturer’s protocol. Cells were washed twice with cold PBS, and CEB-A was added to the cell culture dish. The cell suspension was collected into prechilled tubes, vortexed for 30 s, and kept on ice for 15 min (vortexing for 15 s every 5 min). CEB-B was added, vortexed for 10 s, and incubated on ice for 1 min. After centrifugation at 12,000*g* for 5 min at 4 °C, the supernatant (cytoplasmic fraction) was collected.

The pellet was resuspended in CEB-A and CEB-B, vortexed for 10 s, incubated on ice for 1 min, and centrifuged at 1000*g* for 5 min at 4 °C and repeated again. After discarding the supernatant, the NEB was added to the pellet, vortexed for 15 s, and incubated on ice for 30 min (vortexing for 15 s every 10 min). After centrifugation at 12,000*g* for 5 min at 4 °C, the supernatant (nuclear fraction) was collected.

For insoluble components, the pellet was resuspended in RIPA buffer (20 mM Tris-HCl, 150 mM NaCl, 1 mM EDTA, and 1% NP-40) and 10% SDS, vortexed for 30 s, incubated at 37 °C with shaking (200 rpm) for 30 min, and sonicated for 5 s. The supernatant (insoluble fraction) was analyzed by Western blot alongside cytoplasmic and nuclear fractions.

### Western blot

Cells were collected and lysed with RIPA buffer containing phenylmethylsulfonyl fluoride (Solarbio Life Sciences, R0020) for 30 min on ice, sonicated three times (5 s each), and centrifuged at 12,000 rpm for 15 min at 4 °C. The supernatant was mixed with 5×SDS-PAGE loading buffer (Beyotime Biotechnology, P0015L), boiled at 95 °C for 15 min, and separated on an 8% SDS-PAGE gel (60 V for 30 min, 120 V for 1 h). Proteins were transferred to NC membranes at 300 mA for 1 h, blocked with 5% nonfat milk in Tris-buffered saline with 0.1% Tween 20 at room temperature for 1 h and probed with primary antibodies overnight at 4 °C. HRP-conjugated secondary antibodies were incubated for 1 h at room temperature, and signals were detected using ECL chemiluminescent substrate kit (Biosharp Life sciences, BL520B).

### 1,6-Hexanediol treatment and recovery

Cells in 35-mm dishes were transfected with pEnCMV-N4BP1-GFP at 50 to 60% confluency. 3% or 5% 1,6-HD was prepared in high-glucose DMEM (with 10% FBS and 1% penicillin–streptomycin) and prewarmed at 37 °C. Confocal microscopy images were taken before treatment. The medium was quickly replaced with 1,6-HD solution, and cells were imaged for 5 or 10 min. After treatment, cells were washed with fresh DMEM and recovered for 100 min before final imaging (Leica, TCS SP8).

### Living cell imaging

Cells in 35-mm dishes were transfected with pEnCMV-N4BP1-GFP at 50 to 60% confluency. Live cell imaging was performed on a confocal microscope (Leica, TCS SP8) at 2-min intervals for 30 min.

### Heat shock treatment

Cells were cultured in 6-well plates in 37 °C incubator. When the cell density reached 60 to 70% of confluence, the 6-well plate was put into a 42 °C water bath for heat shock. Cell death was observed in a certain time by an optical microscope.

### Data analysis

Immunofluorescence images were quantified using ImageJ. Western blot data were analyzed with Image Lab. Statistical analyses were performed using GraphPad Prism 10.

## Data availability

All data generated or analyzed during this study are included in this article and the supporting information files. Any additional data and original data presented in this article are available from the corresponding author upon request.

## Supporting information

This article contains supporting information.

## Conflict of interest

The authors declare that they have no conflict of interest with the contents of this article.

## Figures and Tables

**Supplemental Figure S6:**
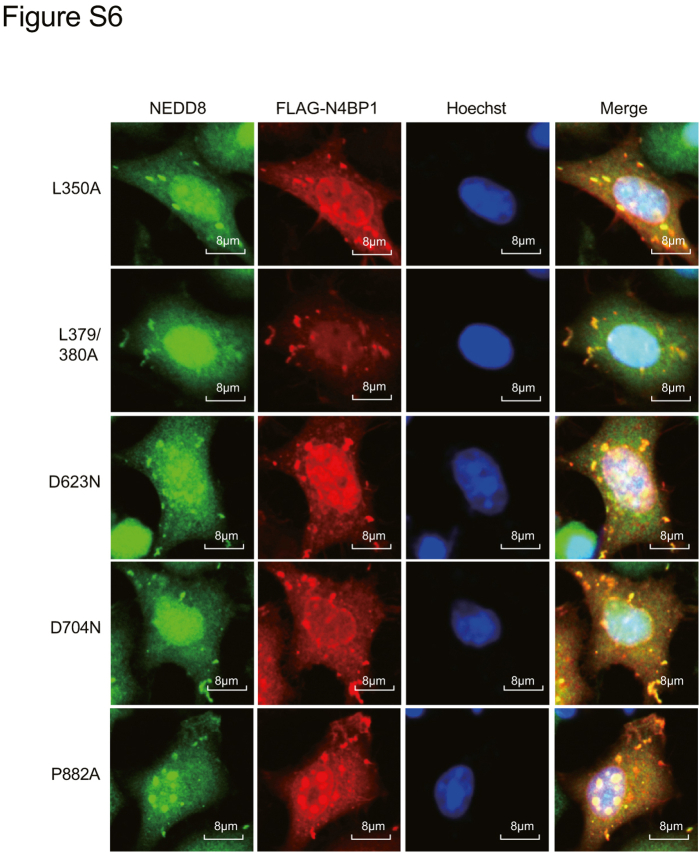


**Supplemental Figure S7:**