# Supplementary material for: N4BP1 is a nucleocytoplasmic shuttling protein and recognizes aggregates of the ubiquitin-like protein NEDD8 to protect cells under heat shock
Source: J Biol Chem. 2025 Jul 21;301(9):110511. doi: 10.1016/j.jbc.2025.110511 (PMC12365338; doi:10.1016/j.jbc.2025.110511)
Supplement: Supplemental Figure legends [file mmc2.docx]

**Supplemental Figure Legends**

**Supplemental Figure S1**

1. FLAG-N4BP1 stably expressed 293T cells were treated with 10 μM Leptomycin B, Importazole or Ivermectin for 2 hrs. The distribution of FLAG-N4BP1 in these cells were determined by immunofluorescence using an anti-FLAG antibody. Images were acquiring using a confocal microscope. Scale bar is 25 μm. (B) Quantification of N4BP1 nuclear-only cells in S1A. N4BP1 nuclear-only cells were counted in 30 cells. The ratio of N4BP1 nuclear-only cells in the 293T FLAG-N4BP1 stable cell line before and after drug treatment is shown. ****, p < 0.0001. Data in (S1A) are representative of three independent experiments.

**Supplemental Figure S2**

1. HeLa cells were transfected with plasmids encoding dKH and NYN-CoCUN and then treated with 10 μM Leptomycin B for 4 h. The distribution of N4BP1 was determined by immunofluorescence using an anti-FLAG antibody. Images were observed by a fluorescence microscope. Scale bar is 10 μm; (B) Quantification of N4BP1 nuclear-only cells in S2A. N4BP1 nuclear-only cells were counted in 30 cells. The ratio of N4BP1 nuclear-only cells in HeLa FLAG-N4BP1-positive cells before and after Leptomycin B treatment is shown. **, p < 0.01; (C) HeLa cells were transfected with plasmids encoding N4BP1-G71D, N4BP1-G93D and then treated with 10 μM Leptomycin B for 4 hrs. The distribution of N4BP1 was determined by immunofluorescence using an anti-FLAG antibody. Images were observed by a confocal microscope. Scale bar is 10 μm; (D) Quantification of N4BP1 nuclear-only cells in S2C and 2E. N4BP1 nuclear-only cells were counted in 50 cells. The ratio of N4BP1 nuclear-only cells in HeLa FLAG-N4BP1-positive cells before and after Leptomycin B treatment is shown. ****, p < 0.0001. Data in (S2A, S2C) are representative of three independent experiments.

**Supplemental Figure S3**

(A) 293T cells were transfected with plasmids encoding GFP or GFP-NLS(279-299). The distribution of GFP was observed by a fluorescence microscope. Scale bar is 10 μm; (B) Quantification of N4BP1 nuclear-only cells in S3A. N4BP1 nuclear-only cells were counted in 30 cells. The ratio of N4BP1 nuclear-only cells in 293T GFP-positive cells after transfection is shown. ****, p < 0.0001. Data in (S3A) are representative of three independent experiments.

**Supplemental Figure S4**

293T cells were transfected with plasmids encoding N4BP1-GFP. Time-lapse imaging captured the disruption and recovery of N4BP1-GFP aggregates following treatment with 3% 1,6-HD. Scale bar is 10 μm. Data in (S4) are representative of three independent experiments.

**Supplemental Figure S5**

293T cells were transfected with plasmids encoding FLAG-N4BP1. The immunofluorescence of CUL1 and CUL2 (green) co-stained with FLAG-N4BP1 (red) and the images were observed by a confocal microscope. Scale bar is 10 μm. Data in (S5) are representative of three independent experiments.

**Supplemental Figure S6**

HeLa cells were transfected with plasmids encoding L350A, L379/380A, D623N, D704N, P822A. The immunofluorescence of NEDD8 (green) co-stained with FLAG-N4BP1 (red) and the images were observed by a confocal microscope. Scale bar is 8 μm. Data in (S6) are representative of three independent experiments.

**Supplemental Figure S7**

1. The control and N4BP1 stably knocking down 293T cells were heated at 42℃ and cell death was observed by an optical microscope. Scale bar is 50 μm; (B) Control and N4BP1 stably knocking down 293T cells were treated or untreated in 42℃ for 30 min. The distribution of N4BP1 were determined by WB after nuclear and cytoplasmic fractionation and insoluble component extraction. Data in (S7A) are representative of three independent experiments. Data in (S7B) are representative of two independent experiments.

**Movie M1**

Time-lapse imaging movie of N4BP1-GFP aggregates in 293T cells. Scale bar is 10 μm. Data in (M1) are representative of four independent experiments.
